# Toward the Effective Bioengineering of a Pathological Tissue for Cardiovascular Disease Modeling: Old Strategies and New Frontiers for Prevention, Diagnosis, and Therapy

**DOI:** 10.3389/fcvm.2020.591583

**Published:** 2021-03-04

**Authors:** Laura Iop

**Affiliations:** Department of Cardiac Thoracic Vascular Sciences, and Public Health, University of Padua Medical School, Padua, Italy

**Keywords:** cardiovascular diseases, disease modeling, *in silico* studies, *in vivo* studies, *in vitro* studies

## Abstract

Cardiovascular diseases (CVDs) still represent the primary cause of mortality worldwide. Preclinical modeling by recapitulating human pathophysiology is fundamental to advance the comprehension of these diseases and propose effective strategies for their prevention, diagnosis, and treatment. *In silico, in vivo*, and *in vitro* models have been applied to dissect many cardiovascular pathologies. Computational and bioinformatic simulations allow developing algorithmic disease models considering all known variables and severity degrees of disease. *In vivo* studies based on small or large animals have a long tradition and largely contribute to the current treatment and management of CVDs. *In vitro* investigation with two-dimensional cell culture demonstrates its suitability to analyze the behavior of single, diseased cellular types. The introduction of induced pluripotent stem cell technology and the application of bioengineering principles raised the bar toward *in vitro* three-dimensional modeling by enabling the development of pathological tissue equivalents. This review article intends to describe the advantages and disadvantages of past and present modeling approaches applied to provide insights on some of the most relevant congenital and acquired CVDs, such as rhythm disturbances, bicuspid aortic valve, cardiac infections and autoimmunity, cardiovascular fibrosis, atherosclerosis, and calcific aortic valve stenosis.

## Introduction

**Figure 1:**
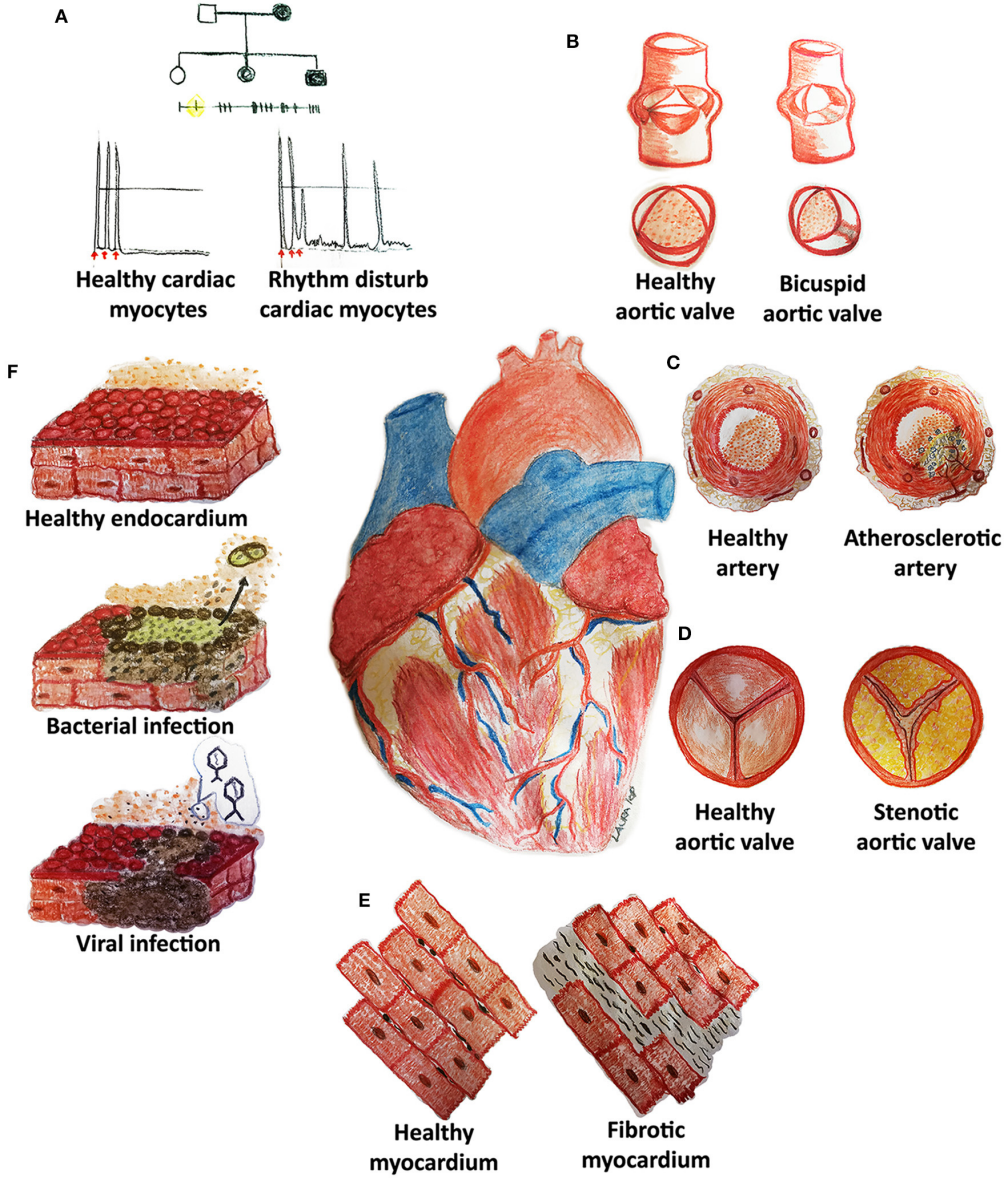
Cardiovascular diseases (CVDs) may affect pediatric and adult patients with congenital **(A,B)** and acquired **(C–F)** pathologies of the heart and its system. **(A)** Rhythm disturbances are caused by mutations of genes codifying, for example, for proteins pivotal in cell electrical activity. **(B)** Bicuspid aortic valves derive from the fusion of two of the three cusps: genetic causes are still not clear although hemodynamic impairment is well-known. **(C)** Atherosclerotic arteries are frequently reported in adult patients and are characterized by an accumulation of lipids (cholesterol), inflammatory cells, endothelial, and smooth muscle cell dysfunction with embolization risks. **(D)** Stenotic heart valves display a fibrocalcific degeneration similar to atherosclerosis. **(E)** Fibrosis may develop in each cardiovascular structure (myocardium, arteries, heart valves) through inflammation and increased collagen deposition. **(F)** Cardiac infections may originate from bacterial or viral contaminations and might cause cell dysfunction and death, thrombi (detachment of dead cells) and other adverse sequelae.

Although the tremendous improvements that modern medicine has witnessed during the last century in terms of prevention, diagnosis, and treatment, cardiovascular diseases (CVDs) still remain the leading cause of death worldwide. Inherited diseases (e.g., congenital rhythm disturbances or extracellular matrix pathologies) as well as acquired forms (e.g., atherosclerosis, thrombosis, fibrosis, infections, malignancies, or cardiomyopathies) represent some of the cardiovascular medical challenges still lacking a complete mechanistic comprehension and/or efficacious treatment (Figure 1) (1, 2).

In this scenario, disease modeling remains crucial to understand the underlying bases and pathognomonic signs, advance novel therapeutic treatments, and propose new preventive actions after a careful evaluation plan. The ability to fully replicate the specific human CVD phenotype is the first step toward an effective modeling process. Therefore, the availability of experimental models able to recapitulate the pathophysiology of CVDs observed in humans is essential in this preliminary phase. So far, different systems have been used for such a purpose with varying ability to reproduce CVD signs. In particular, animal models, especially mammalian ones, have found extensive utilization in the past and remain still the most largely employed tool to study CVDs in the preclinical settings *in vivo*. This experimental modeling can be achieved with animals naturally suffering from a CVD. Also, transgenics and/or toxic stimuli might be applied to artificially develop the diseased phenotype in experimental animals through genetic and/or epigenetic modifications (3–6). Besides animals, mathematical and bioinformatic models have been advanced to simulate *in silico* the typical phenotypic properties of a healthy heart as well as the signs of its dysfunctioning (7, 8). Mammalian cells and/or heterologous expression systems, based on yeasts or Xenopus oocytes, have also found extensive use in the preclinical research on CVDs (9, 10). More recently, pluripotent stem cell technologies have been significantly established as tools to mimic *in vitro* congenital CVDs and hypothesize new therapies (11, 12).

This review describes some examples of CVDs preclinically studied so far by highlighting the advantages and limitations of the used modeling systems and depicting an emerging approach, i.e., the application of tissue-engineered constructs to simulate more appropriately human cardiovascular pathologies (13).

## *In Silico, In Vivo*, and *In Vitro* Modeling: Opportunities and Challenges for CVD Recapitulation

**Figure 2:**
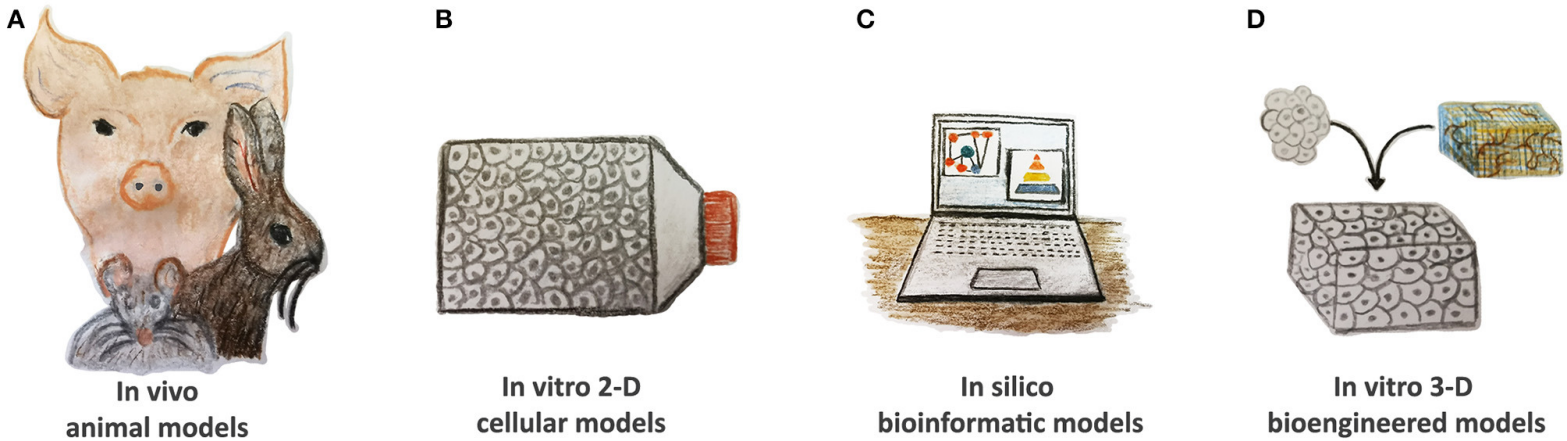
Current tools of CVDs modeling. **(A)** *In vivo* animal models, such as rodents, rabbits, and pigs, have been widely used to study CVD pathomechanisms; however, they display many anatomical and physiological differences when compared to humans. **(B)** *In vitro* two-dimensional (2-D) cellular models based on animal and human cells as well as on pluripotent stem cells demonstrate their validity to evaluate the altered pathway at the single-cell level. **(C)** *In silico* bioinformatic tools gather together all the information and knowledge generated in other models, consider stratification risks, and advance new hypotheses. Reproducible validation is compulsory. **(D)** *In vitro* 3-D bioengineered models are gaining more interest in the cardiovascular research community by offering human platforms for CVD modeling in precision medicine settings. They might also be applied to verify the therapeutic hypotheses of *in silico* simulations.

An ideal disease model should completely replicate human pathophysiology with the vital goal to provide relevant scientific knowledge to be translated into clinical practice for effective health care management. As such, cost-effectiveness, easy manipulation, and adequate statistical power are essential selection criteria. CVDs are a very heterogeneous class of congenital and acquired pathologies. They often display complex molecular interplay, phenotype, and symptomatology by offering many challenges to effective modeling. Several approaches are found to be useful to reproduce CVDs based on *in silico, in vivo* animal, *in vitro* two-dimensional (2-D) cellular, and three-dimensional (3-D) bioengineered models (Figure 2).

*In silico* tools, such as mathematical and bioinformatic simulations, are gaining more and more interest because they provide the possibility of implementing all possible variables playing in a disease. Bioinformatics is a scientific field gathering together multidisciplinary approaches (biology, medicine, chemistry, physics, engineering, etc.) to analyze data observed experimentally and/or clinically. By this investigation, a computational model can be built to describe the biological phenomenon and its perturbations opportunely. In particular, this modeling modality offers valuable insights when coupled with -omics technologies. The computational analysis of raw data generated from whole genome, proteome, and metabolome evaluations has further contributed to profoundly investigate the disease alterations of biological systems or physiological functions. Such modeling proceeds through data mining and machine learning (14). It is extensively used to advance predictive CVD models by integrating information generated across multispatial scales. For example, it may allow studying the behavior of cardiac ion channels and other proteins in the context of the cell membrane (often coupled with heterologous expression systems *in vitro*). Moreover, it can reconstruct the pathomechanism leading to the onset or progression of a specific CVD, or it can contribute to clarifying the altered dynamics of blood flow and/or pressure in a dysfunctional cardiovascular region (15–17). Nevertheless, it requires extensive comprehension of the considered pathology and its events to advance their bona fide replicas through algorithms. It needs robust computational systems to run high-throughput simulations, and it must rely on credible experimental verifications (18).

Modeling with mammals and also with other animals (fish, for instance) has been the most used strategy throughout biomedical history: relatively low costs and easy handling are advantageous, remarkably for smaller size organisms. Thanks to the advent of transgenesis, mice have become the mammals of choice to study human CVDs. Haploinsufficiency, knockdown, and insertions are only a few of the genetic mutations introduced into the mouse's genome for its humanization. Less frequently, rats, hamsters, and guinea pigs, eventually genetically engineered, are serving as models, too. The widespread use of rodents in experimental research enabled the dissection of the intricate molecular pathways of congenital and acquired CVDs. However, it is noteworthy to consider that rodents display many anatomical and physiological differences from humans (e.g., abundant collateral circulation, particularly in the heart, increased beating rate, reduced inflammation, absent stenosis) (19, 20). Dissimilarities with human anatomical and metabolic properties also exist with other animals, among which are rabbits, dogs, cows, pigs, and seldomly non-human primates (19, 20). These animals are more expensive and difficult to handle due to their size, but they are phylogenetically closer to the human species. *Per se*, no animal can work as a perfect model to recapitulate human CVD. However, it offers the unique opportunity to gain more knowledge of the peculiar pathology *in vivo* in a dynamic, open system considering both the diseased tissue (or organ) and the organism's response.

*In vitro* experimental, investigational tools based on cells are frequently applied to complement animal-based studies. They allow focusing on a low number of variables and are principally utilized to evaluate the contribution of a single pathway or cell type alteration in a given pathology. *In vitro* cellular models have been developed with differentiated animal and human cells and, more recently, with induced pluripotent stem cells (iPSCs) (21). This latter option derives from the reprogramming to pluripotency of somatic cells, harvested by human patients harboring a genetic disease or by healthy subjects, considered as controls. The virtually unlimited ability of iPSCs to differentiate in all cell types of the three germ layers has offered an unprecedented modality to study diseases in a Petri dish. As adult human cardiomyocytes, several cell types are often difficult to obtain by primary culture or might show instability *in vitro* (15). When dealing with primary lines, one of the significant drawbacks of cell culturing is represented by their instability, together with the risk of enriching cell types not representative of a whole heterogeneous tissue population. Regarding human iPSC-based platforms, incomplete differentiation may fail to uncover the involved pathways and phenotypical hallmarks of the real pathologic settings.

The classic 2-D cellular modeling has been surpassed in most of its limitations by 3-D. Mimicking *in vitro* the complex interplay of cells and extracellular matrix can considerably improve the ability to reproduce the typical disease signs observed in the clinic. The application of bioengineering principles has lately demonstrated its relevance in this context (22). Tissue engineering and regenerative medicine stemmed as a multidisciplinary approach to heal diseased tissues and organs and restore homeostasis. By opportunely combining natural or synthetic scaffolds with specific cell types, tissue engineering aims to recreate *in vitro* tissue equivalents (23). The extracellular matrix can be reproduced artificially by an assembly of synthetic polymers or obtained naturally by manipulating human and animal native tissues, for example, by decellularization (24, 25). 3-D bioengineered constructs reproducing the tissue or organ of interest find increased utilization to simulate physiologic and pathologic settings *in vitro* (26–28). They cannot be considered an open system such as the entire organism. However, they might investigate dynamic scenarios by enclosing other players, such as biochemical stimuli, biomechanical conditioning, different cellular types, etc., in bioreactor platforms (13).

## Modeling of Congenital Heart Diseases

Congenital CVDs can be subdivided into two main categories, underlying structural or non-structural defects. The bicuspid aortic valve, catecholaminergic polymorphic ventricular tachycardia, and arrhythmogenic disease are inherited pathologies associated with mutations of one or more genes pivotal for cardiovascular function.

### Structural Defects

#### Bicuspid Aortic Valve

##### Epidemiology, Clinical Presentation, and Underlying Causes

Among the structural malformations affecting the heart and its vessels, a bicuspid aortic valve (BAV) is the most commonly observed in the general population with an overall incidence of 1–2% and male prevalence (29, 30). It can be associated with diseases of the aorta and other valves by being often encountered in several complex syndromes (31). It results from the anatomical fusion of two cusps in an otherwise normal three-leaflet valve of the left outflow tract, consequently altering aortic hemodynamics.

BAV presentation may differ individually in complexity and forms (isolated or syndromic). It can also show an inheritance pattern typical of a dominant genetic disease with variable penetrance (31). The underlying mechanistic cause is largely debated. From valve formation to its calcific evolution, BAV tissues may display alterations in several pathways and biological functions. Among the latter, abnormal endothelial to mesenchymal transition (endMT) and neural cardiac crest cell behavior as well as extracellular matrix dysregulation and altered metabolism of nitric oxide have been frequently observed (30, 32). The NOTCH family was one of the first gene candidates to be tested as causative. Its signaling is involved in embryonic valve formation. Any genetic perturbation affects the extracellular matrix's normal deposition and other functions, such as apoptosis and bone development (33, 34). The NOTCH1 non-sense mutation is found in some BAV pedigrees. However, its association remains with sporadic, familial cases of isolated disease (35, 36). Linkage analyses performed on BAV syndromic families reveal the co-involvement of other genes, such as ACTA2, codifying for smooth muscle actin, and TGFBR 1 and 2, encoding the cell receptors of the TGF beta family (37). Interestingly, genetic variants of ROBO4, a gene fundamental for endothelial cell function, have been found to segregate with BAV in a study of two families (38). However, no etiologic knowledge is evident yet despite the use of the most recent high-throughput gene sequencing technologies.

##### Animal Models

The comprehension of BAV has been rendered challenging by the difficulty of modeling the disease in experimental settings. Few models are currently available to study BAV development and progression. Nigam and Srivastava described in 2009 a higher propensity to aortic valve cusps thickening and calcification in NOTCH1 haploinsufficient mice (Notch 1^+/−^). They reported no other BAV signs. Besides this *in vivo* animal model, they also confirmed through an independent *in vitro* study the pro-calcific effect of Notch pathway inhibition on sheep normal aortic valve interstitial cells (VICs) (39).

NOTCH mutations were not the only ones disclosed for BAV-presenting animals. In the study by Fernández et al. (40), adult eNOS knockout mice, i.e., deficient in endothelium nitric oxide synthase, and inbred Syrian hamsters displayed BAV with incomplete penetrance as well as differential cusp involvement. It was revealed that eNOS deficiency in mice induced an altered endMT and was responsible for the fusion of the right and non-coronary cusps. In hamsters, the left and right cusps were found fused in association with altered neural crest cell behavior. In another publication, the same group re-confirmed these valve findings in the hamster and described other signs, such as cranial defects (41).

A potentially adverse effect on crucial valvulogenic processes was hypothesized as being caused by altered neural crest cells (32). The haploinsufficiency of Nkx2.5, a cardiac homeobox gene, revealed an 8-fold increased propensity toward BAV development in the experimental model (42); yet, it has not been described as associated with humans (43).

Generally, animal models show few of the phenotypical signs typically observed in BAV patients. Besides the causes, the effects have been carefully investigated by deriving BAV calcification and stenosis animal models. Calcification frequently occurs in BAV subjects with a chronic progression, more rapid and adverse than in patients with tricuspid aortic valves (44). Calcific aortic valve stenosis modeling is addressed with more attention in the following section dedicated to acquired diseases.

##### *In silico* Models

Stenotic consequences have also been modeled numerically in biomechanical analyses of bicuspid valves. Finite element studies based on *in vivo* magnetic resonance imaging data have reconstructed BAV biomechanics *in silico*. Conti et al. revealed abnormal cusp stress (+800%) in their analysis performed in 2010 (45). They hypothesized this as having a role in the globally altered behavior of the diseased aortic valve. High stress was demonstrated at the aortic wall, too, especially in aneurysmatic conditions. In 2014, Forsell et al. compared collagen fiber distribution and stiffness, related either to collagen or elastin, in aneurysmatic aortic wall rings excised surgically from patients with tricuspid and bicuspid aortic valves (46). They revealed that only collagen-related stiffness and, hence, strength significantly increased in BAV patients and proposed applying these simulations as complementary to the molecular investigation. Simulation of fluid dynamics derived from 3-D computed tomography angiography of BAV patients evidenced anomalous helical flow through the aorta, characterized by impingement at the level of its proximal ascending wall. This computational reconstruction made it possible to appreciate the augmented shear stress at the interested wall area due to the abnormal flow (47). Apart from aortic jet flow, BAV fluidics is also characterized by more turbulent, oscillatory shear stress in the calcified, fibrosal cusp regions (48).

Interestingly, the advent of 3-D printing technology has brought more ease in modeling BAV aortic hemodynamics. Gill et al. recently replicated BAV valve cusps in a custom aorta mold and studied flow characteristics through 4-D magnetic resonance imaging. Simulations obtained by collected data can serve as more accurate tools to estimate the transvalvular pressure drops, often masked by the turbulent aortic flow jet (49). Moreover, it is increasingly applied for the planning of effective transcatheter replacement procedures (50).

##### 2-D Cellular Models

Godby et al. developed an *in vitro* dynamic cellular model to evaluate the influence of BAV altered biomechanics on VICs (51). As previously demonstrated by Nigam and Srivastava in sheep VICs (39), healthy human counterparts underwent phenotypic changes with nodule formation upon *in vitro* inhibition of the NOTCH pathway (51). In addition, by applying a low fluidic oscillatory shear stress, healthy and BAV VICs expressed lower levels of ACTA2 and ELN (elastin gene). NOTCH pathway inhibition in dynamic conditioning had two main consequences. First, only ELN transcripts resulted in being decreased in BAV VICs. Second, a smooth muscle actin-positive subpopulation of myofibroblasts became enriched. The authors postulated such a myofibroblast increase as the possible mechanism for the onset of BAV calcification observed *in vivo*.

The crosstalk between biomechanics and molecular signaling is nowadays an established concept and is at the basis of the effects that the extracellular environment, matrices and scaffolds included, exerts on the cells immersed in it. Novel BAV modeling approaches should more accurately consider the strict correlation between biomechanics and cell behavior. Hence, they should implement all the factors triggering the pathological valve phenotype of affected patients.

### Congenital Rhythm Disturbances

Rhythm disturbances represent a particular class of congenital diseases. Affected patients may present a structurally normal heart but develop abnormal electric cardiac activity in response to specific triggers. This class of pathologies is generally rare but heterogeneous. Cardiac rhythm pathophysiology often occurs due to mutations in genes codifying for ion channels. Some examples of rhythm disturbances are catecholaminergic polymorphic ventricular tachycardia (CPVT), long QT (LQT), and arrhythmogenic cardiomyopathy (AC).

In the past, the modeling of these diseases was mainly performed through animals. Transgenic mice were generated by introducing a targeted mutation of the disease genes homologous to reproduce the human genotype. Additional approaches have also been followed to integrate with further molecular studies and/or computational simulations (15).

#### CPVT

##### Epidemiology, Clinical Presentation, and Underlying Causes

CPVT manifests clinically with supraventricular arrhythmias after physical/emotional stress or catecholaminergic surges. Disease prevalence is still unpredicted; among diagnosed patients, about 30% are not responsive to pharmacological treatment and may undergo fatal arrhythmias (52).

Several CPVT forms were described as depending on the mutation of different genes. RYR2, CSQ2, and TRDN, respectively, encoding for ryanodine receptor 2 channel, calsequestrin 2, and triadin, were reported as mutated. Despite the heterogeneous gene involvement, the resulting phenotype is a defective calcium handling. Indeed, all these genes codify for proteins that possess an essential function on calcium regulation in cardiomyocytes. When the RYR2 gene is mutated, the spontaneous release of calcium from the sarcoplasmic reticulum due to its store overload is a typically observed phenomenon, which induces arrhythmic events, such as after depolarizations and triggered activities (11, 53).

##### *In silico* Models

Successful analyses of multisubunit channel functionality in CPVT were performed using *in silico* models simulating the sarcoplasmic membrane or based on *in vitro* heterologous cell systems (54–57). Based on these studies, RYR2-related CPVT was hypothesized as induced by two possible mechanisms explaining the calcium-leaking behavior of the defective channel. An inability to bind to the accessory protein FKBP12.6 has been postulated as the reason for the calcium channel's instability (58, 59). Alternatively, a loss of subunit interaction or domain unzipping in the ryanodine receptor 2 heterotetrameric complex has been suggested to alter calcium homeostasis (54).

##### Animal Models

An animal study based on a RYR2 knock-in mouse demonstrated that a gene mutation was unlikely to induce an ineffective protein–protein binding. No alterations were observed in the binding between the RYR2 channel and FKBP12.6, either at rest or after epinephrine or caffeine stimulation (60).

##### 2-D Cellular Models

Results from a patient-specific iPSC-based *in vitro* model by Jung et al. support domain unzipping as a plausible cause of abnormal calcium release in cardiomyocytes with defective RYR2 (11). This study demonstrated for the first time that pharmacological treatment with dantrolene can effectively stop arrhythmic events generated by catecholaminergic stimulation. The dantrolene-induced phenotypic rescue was later confirmed by other *in vitro* iPSC models and in clinical patients carrying RYR2 mutations (EudraCT Clinical trial) (61).

Other drugs were tested in similar settings for the treatment of CPVT. Enhancement of mitochondrial calcium uptake by efsevin was demonstrated as a valid strategy to abolish arrhythmias in an *in vitro* model based on human CPVT iPSC-derived cardiac myocytes and *in vivo* in a transgenic mouse carrying a human typical disease mutation (62). Pölönen et al. tested *in vitro* the antiarrhythmic activity of carvedilol and flecainide. These drugs are known beta-blockers administered clinically to treat chronic heart failure and CPVT. For this purpose, three iPSC lines were used: two generated from CPVT patients with different mutations and one from a healthy subject. Under adrenergic stress, calcium transient abnormalities manifested in CPVT iPSC-derived cardiomyocytes but could be suppressed by tested drugs although with varying degrees of efficiency (63). Similar results are also shown by Maizels et al. in another *in vitro* model based on iPSCs displaying other CPVT mutations (64). Remarkably, flecainide was able to inhibit calcium alternans abnormalities (63, 65). Characterized by alternating amplitude patterns, cardiac alternans are not life threatening but can be considered a risk stratification marker of the most malignant CPVT forms (66). Due to their relevance in predicting disease severity, *in silico* computational models have been designed to hypothesize inducing mechanisms and abolishing modalities (67).

##### 3-D Tissue-Engineered Models

*In vitro* CPVT modeling has recently gained more information by coupling iPSC technology with heart tissue engineering. Park et al. (68) developed a microphysiological model of CPVT to reproduce the substrate conditions for reentrant or focal arrhythmias observed in the working myocardium. This tissue was recreated *in vitro* by combining gelatin chips and three different lines of CPVT iPSC-derived cardiac myocytes (iPSCs derived from a healthy donor were used for controls). In the settings of adrenergic stimulation or increased pacing, this platform recapitulated the spiral wave reentry, which is the typical cause of tachycardia events in the heart tissue of diseased patients. This behavior was only prominent for CPVT engineered myocardial tissues and was abolished by dantrolene by confirming previous observations collected in the 2-D modality (11, 61). The 3-D CPVT model was revealed to be substantially superior to the 2-D conditioning for its ability to increase maturation and stabilize the electrical activity of iPSC-cardiomyocytes at rest (68).

A drug-testing platform based on CPVT cardiac-engineered tissue was proposed by Goldfracht et al. (28). A hydrogel derived from decellularized porcine heart extracellular matrix was enriched with chitosan and used as a scaffold to reconstruct myocardium with cardiac myocytes differentiated from one CPVT, one long QT, and one healthy iPSC lines. The enriched hydrogel efficaciously contributed to rendering more differentiated and mature cardiac myocytes derived from iPSCs in all lines tested. At high pacing frequency, reentrant arrhythmias were effectively reproduced. Several drugs, such as lidocaine, an antiarrhythmic agent, carbenoxolone, a gap junction blocker, quinidine, a sodium channel inhibitor, and/or dofetilide, another antiarrhythmic compound, were administered under stress and confirmed the effects observed in the clinical treatment of patients. The application of a prolonged electrical field potential could also restore normal electrical behavior, thus experimentally simulating the electrical cardioversion clinical procedure.

#### AC

##### Epidemiology, Clinical Presentation, and Underlying Causes

Another challenging congenital disease in the clinical practice is represented by AC, a rhythm disturbance with right ventricle or biventricular structural dysplasia. The hearts of suffering patients (1:2000–1:5000 prevalence) may display fibrofatty deposits, generally in the right part. These interfere with the conduction system and become a substrate for non-ischemic ventricular arrhythmias and, ultimately, sudden death. This disease has been associated with inherited autosomal mutations (predominantly dominant) in genes encoding for junctional proteins (plakophilin-2, plakoglobin, desmoplakin, desmogleins, etc.), their accessory components or associated nuclear membrane proteins (TMEM transmembrane protein, TGFβ 3, and β -catenin) (69).

The mechanism by which cardiac muscle is replaced by fat and fibrous tissue is still poorly understood, and various hypotheses have been formulated, including increased ketogenesis and mitochondrial dysfunction (70). Due to the phenotypic complexity of AC disease, several factors are thought to contribute to its pathomechanism.

##### Animal Models

Heterologous expression systems were initially useful to understand the defective desmosome developed by mutated gene products (71). AC modeling has been mainly performed through animal studies. Like humans, boxer dogs may develop AC spontaneously when they are natural carriers of mutated desmosomal proteins (72).

Transgenesis strategies have been applied to generate AC mouse models (69). Although these mice carry the same mutations observed in boxer dogs and humans, their phenotype is not identical: no fat infiltration is generally observed in the rodent heart myocardium. In addition, transgenesis techniques are more successful in reproducing loss of function than missense mutations (73).

##### 2-D Cellular Models

Cellular models generally complement or stem from *in vivo* studies to closely investigate AC signs and mechanisms at the single-cell level. For example, cardiac progenitors derived from a plakoglobin-mutated mouse model showed a facilitated conversion to adipocytes by Lombardi et al. (74). As such, a transdifferentiation cell event could be the mechanism by which the AC phenotype manifests. The defective plakoglobin is not able to correctly assemble at the desmosomal junction. Hence, it could remain unbound and translocate to the nucleus, where it may compete with β-catenin for the activation of the T-cell factor/lymphoid enhancer binding factor. This series of molecular events could lead to the activation of the fat transcriptional program at the muscular one's expense, thus transforming a myocyte into an adipocyte. This possible impairment of the Wnt pathway could be a viable hypothesis for AC pathomechanism. Cardiac progenitor cell conversion into the adipocytic lineage is demonstrated to be reversed *in vitro* by GSK-3β inhibitor, i.e., a Wnt signaling activator (74).

iPSC-cardiomyocytes derived from clinical patients harboring plakophilin 2 gene mutations are proven to be useful tools to effectively model human AC (12, 75–79). In the study by Caspi et al., the upregulation of pro-adipogenic transcription factor peroxisome proliferator-activated receptor gamma (PPAR-γ) and other adipogenesis-related genes was confirmed by the phenotypic accumulation of lipid droplets in iPSC-cardiomyocytes. As previously demonstrated for cardiac precursors isolated from transgenic mice, the Wnt pathway reactivation by the GSK-3β inhibitor was sufficient to reverse the pathophysiological phenotypic changes (75), proving once again the significance of the *in vitro* iPSC-based models in drug testing. Furthermore, Kim et al. showed that apoptotic events and abnormal calcium handling could also manifest. They observed exaggerated lipogenesis and revealed that a metabolic change from an embryonic glycolytic to an adult-like energetic state is necessary for AC development (77).

More recently, Dorn et al. evidenced the importance of cell–cell and cell–matrix contacts in regulating development and lineage specification. In the AC setting, the cell adhesion weakening favors the differentiation of cardiac myocytes into brown/beige adipocytes. By combining *in vitro* studies based on iPSCs, embryonic stem cells, and cardiac mesenchyme to *in vivo* lineage tracing, they also investigated the link between embryonic development and disease pathomechanism. The prominent involvement of the right ventricle underlies the transdifferentiation proneness of a specific set of “myo-adipo progenitor cells,” which originates from the secondary heart field and expresses the homeobox genes Isl1 and Wilms tumor 1 (Isl1^+^ Wt1^+^ cells) (12).

As observed by Martewicz et al. (79), mechanical stress dramatically influences the response of AC iPSC-cardiomyocytes grown on patterned surfaces: a strong activation of fibrosis pathways but, unexpectedly, not of adipogenic ones, was revealed in their model.

##### 3-D Tissue-Engineered Models

Bioengineering a tissue to mimic AC is in its initial stage. Nevertheless, it provides clear insights into the influential relationship between cells and the extracellular matrix in this congenital rhythm disease. A decellularized cardiac scaffold served Tung et al. to develop a tissue-engineered AC heart model. The combination of iPSC-derived cardiac myocytes and a cellular scaffold allowed recapitulating *in vitro* the 3-D complexity of AC: cellular alignment, tissue architecture, and lipid accumulation. It also increased cell maturity in myocytic structure, ion channel repertoire, junctional proteins, and calcium handling, which are challenging to reach through 2-D culturing. Three-dimensionality also augmented metabolic and apoptotic cell functions. Sustained reentrant activities were observed in AC-engineered heart tissues upon pacing and resulted in being exacerbated by a gap junction blocker (80).

## Acquired Heart Diseases

Several CVDs may develop during a subject's lifetime as associated with many factors, including lifestyle, genetic predisposition (non-pathogenic polymorphisms), and/or external triggers. Most of these diseases, e.g., calcification, fibrosis, and atherosclerosis, have a chronic progression that is often severe. Others, such as infections, are mainly acute pathologies, whether adequately diagnosed and treated, but can also cause heart failure by induction of autoimmunity (81).

### Cardiac Infections and Cardiac Autoimmunity

#### Epidemiology, Clinical Presentation, and Underlying Causes

Infections, especially bacterial, have represented a severe therapeutic challenge in the past for what are now industrialized countries. They can still be considered a medical threat in the underdeveloped world. Antibiotic treatments have entirely revolutionized the history of bacterial infections. These pharmacological therapies have drastically reduced the adverse effects of associated rheumatic fevers on the heart, especially on its valve apparatus and myocardium (82, 83). The sequelae of group A β-hemolytic *Streptococcus pyogenes* pharyngeal infections cannot be effectively prevented in developing countries due to indigence, inadequate hygienic conditions, and/or poor therapeutic compliance but they could be defeated by a specific vaccine, claimed for treatment but still not available (84).

Rheumatic fevers develop in the pathophysiological response to bacterial and also to viral infections. Viral cardiac diseases are mostly triggered by coxsackieviruses, adenoviruses, parvovirus B19, human herpesvirus-6, and more recently by Sars-CoV-2 (COVID-19) (85).

Autoimmunity is often a common-denominator mechanism in the reaction cascade to bacterial and viral microorganisms. It is predicted to arise from an erroneous recognition of endogenous epitopes as non-self due to their resemblance to proteins belonging to pathogenic organisms (82, 86). Consequently, the immune system is likely to be cheated by the microorganism's mimicry strategies to increase its survival in the host through cross-reactive antigens. Of note, rheumatic heart disease may generate subsequent infections with cardiac tropism and also as a consequence of other autoimmune diseases, such as systemic lupus erythematosus, rheumatoid arthritis, and systemic sclerosis (87).

#### Streptococcal Infections

##### Animal Models

Experimental modeling of cardiac infections is crucial to understand the fundamental mechanism underlying autoimmunity development and also to evaluate possible therapeutic targets and pharmacological treatments. The recapitulation of the human cardiac pathophysiology of *Streptococcus pyogenes* using animals failed. Animals generally display a limited susceptibility to this pathogen and a fast resolution of induced infection (84) apart from a specific rat strain. Lewis rats immunized with peptides of the conserved region of streptococcal M protein developed antibodies against this last epitope and cardiac myosin. Hearts isolated from this autoimmune model showed the typical signs of rheumatic disease, including valvulitis and myocarditis (88). More recently, in a similar rat model, Chen et al. demonstrated that miR-155-5p inhibition of S1PR1 and SOCS1 genes, respectively, encoding for sphingosine-1-phosphate receptor 1 and suppressor of cytokine signaling 1, abolished the autoimmunity and rheumatoid effects of streptococcal infection (89).

##### *In silico* Models

*In silico* modeling of rheumatic heart disease induced by *Streptococcus pyogenes* infections might be considered more helpful in the design of possible therapies. In particular, reverse and structural vaccinology approaches have the potential to develop successful vaccine hypotheses. For such an aim, novel gene candidates could be explored in the whole bacterial genome. Moreover, conformational studies of streptococcal epitopes could help to design a vaccine based on a multidomain antigen molecule (84).

#### Sars-CoV-2 Infection

Sars-CoV-2 infection represents the most current menacing, worldwide health emergency. The COVID-19 pandemic is characterized by a series of clinical signs that can reach, in the worst evolution scenario, a fatal severe acute respiratory syndrome, too. Its symptomatology was initially compared to SARS due to the pulmonary involvement. However, during its spreading throughout the world and the medical observation of its progression, it appears more evident that the lungs and other organs, heart included, are targeted by this infection. Viral myocarditis, arrhythmias, and ultimately heart failure are among the clinical signs reported (90, 91).

The sudden clinical appearance of COVID-19 has found the biomedical community unprepared to face this infection's fast and severe evolution. It has boosted the research to find effective disease modeling platforms and therapeutic strategies urgently. Little information was initially available on the virus, apart from its family, i.e., coronavirus, similar to SARS and other viruses inducing seasonal cold. A spike protein guarantees its viral entry ability thanks to the affinity for the host's angiotensin-converting enzyme 2 (ACE2) (92–94). The multicellular expression of this protein justifies the multiorgan tropism shown by this virus.

##### 2-D Cellular and 3-D Tissue-Engineered Models

*In vitro* infection studies with several human and animal carcinoma cell lines—including pulmonary Calu3, colorectal Caco2, and cervical HeLa adenocarcinoma cells—demonstrated their essentiality to understand the mechanism of the viral entry (91, 92), but they could less efficiently reproduce the *in vivo* post-entry phase in normal human tissues.

The pluripotency of human iPSCs is a powerful tool for such a task. It shows usefulness *in vitro* to evaluate the organ-specific cellular events induced by this pandemic virus. iPSC-derived platforms are applied with high reproducibility and throughput. Yang et al. differentiated human iPSCs in the three germ layers and generate pancreatic endocrine cells, liver organoids, endothelial cells, cardiomyocytes, macrophages, microglia, and cortical and dopaminergic neurons. Upon inoculation with the Sars-CoV-2 virus, the most infected iPSC-derived cells were pancreatic cells, liver organoids, cardiomyocytes, and dopaminergic neurons. Moreover, a higher permissiveness to viral entry was confirmed in adult primary cholangiocyte and hepatocyte cell lines (95). These preclinical outcomes are consistent with the higher expression of ACE-2 shown by these cells and recapitulate the clinically observed affection of lungs, gastrointestinal system, heart, and brain in diseased patients.

More hints on the mechanism and effects of Sars-CoV-2 infection on cardiac myocytes are proposed through two recent *in vitro* studies based on human iPSCs (96, 97). In particular, Sharma and colleagues described the entry and replication ability of the Sars-CoV-2 virus in infected iPSC-derived cardiomyocytes. Perinuclear localization, contractility loss, apoptosis, and death were observed within a time-lapse of 72 h (96). Kwon et al. showed that extracellular vesicles from Sars-CoV-2-infected lung epithelial cells can behave like a virus vehicle for iPSC-cardiomyocytes (97).

##### *In silico* Models

*In silico* models of the spike interaction for the viral entry have been developing, too. Combined with *in vitro* 3-D hiPSCs-based studies, they are impressively facilitating the successful testing of drugs, promptly applied in the clinics for the therapy of infected patients (98).

#### Bacteria- and Virus-Based CVD Modeling Approaches for Imaging and Treatment

Apart from infection disease modeling, the peculiar cardiac tropism shown by some pathogenic microorganisms can be exploited to target imaging and therapies. Several strains of *Escherichia coli* and *Salmonella typhimurium* exhibit selectivity for the heart muscle rather than for non-cardiac tissues. Le et al. took advantage of this bacterial strain's ability to target infarcted myocardium in a rat model through a defective *S. typhimurium* strain, engineered to express an inducible luciferase gene. These genetically engineered *Salmonella* cells were infused in the tail vein of the rat. Upon luciferase induction, bioluminescence imaging confirmed bacteria localization in the infarcted heart (99). Bacterial strains with cardiac tropism could be attenuated and engineered as a vehicle for gene or drug therapies. Such a therapeutic approach will need further investigation in terms of pathogenicity, even if used bacteria are attenuated.

Similar to defective bacteria, attenuated viruses with cardiac tropism have been proposed as possible vehicles for gene therapies or *in vivo* bioengineering strategies. Direct cardiac reprogramming based on retroviral vectors was demonstrated by Qian et al. (100, 101). By local injection of attenuated retroviruses engineered to express GATA4, MEF2c, and TBX5, cardiac fibroblasts were directly induced to cardiomyocytes in a model of rat myocardial ischemia (101). As for any bacteria-based treatment, therapeutic strategies relying on viral vectors require extreme caution, especially considering reactivation risks.

### Cardiovascular Fibrosis

#### Epidemiology, Clinical Presentation, and Underlying Causes

Fibrotic tissue results from an unsuccessful attempt by the body to repair after an acute or chronic insult (mechanical damage, autoimmune response, infections, etc.). In the normal process of wound healing, damaged epithelial/endothelial cells secrete inflammatory cytokines that contribute to platelet activation and fibrin clot formation. Therefore, a lymphoproliferative response is started with leukocytes recruited to the injury site and releasing profibrotic chemokines, as TGF-ß. Consequently, damaged cells and myofibroblasts—possibly differentiated from circulating mesenchymal stem cells or transdifferentiated from epithelial/endothelial cells—are stimulated to secrete metalloproteinases. These released enzymes digest the basal membrane. The secretion of other cytokines enables the recruitment and activation of neutrophils, macrophages, T and B cells, and eosinophils. During debris removal by macrophages and neutrophils, myofibroblasts synthesize a new extracellular matrix, invaded by vessels newly constituted by endothelial cells. Wound contraction is initiated by activated myofibroblasts, causing collagen reorganization, blood vessel loss, scar elimination, epithelial/endothelial cell proliferation, and migration onto the reconstituted basal membrane. Such a return to healthy tissue is not achieved when inflammation remains unresolved. In such a condition, a persistently active myofibroblast population dedicates to extracellular matrix secretion and ultimately starts fibrosis (102).

As recently reviewed, fibrosis is an age-related disease and may interest the cardiovascular system and other organs (103). Fibrosis occurs in several pathologies involving the heart and its structure, such as valvulopathies, hypertension, arrhythmias, myocardial ischemia, and heart failure, and often contributes to their severe clinical course. So far, no efficacious therapy to prevent its development and sequelae is available (104–106). The peculiar characteristics of the fibrotic process affecting the cardiovascular system is described in the following sections dedicated to heart, valves, and arteries.

#### Fibrosis in the Heart

In the fibrotic heart, cardiac fibroblasts acquire a contractile and migratory myofibroblast phenotype very quickly, as proposed by Berk et al. (104). This transition is significantly regulated by endothelin 1 and angiotensin II, which, in turn, contribute to activate TGF-ß. Consequently, cardiac myofibroblasts initiate a sustained profibrotic program, especially in the hypertensive heart. The extracellular matrix is abnormally synthesized and only partially cross-linked. An anomalous deposition occurs for several proteins, such as collagens I and III, elastin and its precursor fibrillin, fibronectin, proteoglycans, and glycosaminoglycans. The degradation of extracellular matrix, controlled by metalloproteinases and their tissue inhibitors, results in impairment, too (107–109). Persistent vascular injury is another characteristic event in heart fibrosis. Apart from TGF-ß, a plethora of pro-inflammatory cytokines circulates during this phase. They contribute to the recruitment of more smooth muscle cells, monocytes, and fibroblasts, and thus, they subsidize a chronic profibrotic state. As a result, stiffness increases by inducing alterations in myocyte cells' contractility and relaxation properties. Modifications of the cardiovascular cellularity, including cardiomyocytes and smooth muscle cells, occur (104). These alterations irreversibly lead to global heart failure (systolic and diastolic).

##### *In silico* Models

Computational modeling of fibrosis in the context of other diseases affecting the myocardium mainly focuses on the effects on blood flow dynamics and electrical activity in global heart function. Such an integrating approach appears essential to stratify the risk of adverse disease evolution in affected patients (110, 111).

At the cellular magnitude, fibrosis modeling has gained more interest in better characterizing involved signaling pathways and identifying effective pharmacological treatments. Activation of endothelial cells during endMT was, for example, simulated computationally by Weinstein et al. (112). A dynamic Boolean model of the pathways involved in the endMT generated a framework easily modifiable to investigate gain- or loss-of-function, disease evolution, and pharmacological targeting.

##### Animal Models

*In vivo* animal models of cardiovascular fibrosis were generated and allowed to increase knowledge on the fibrotic process's evolution. Simulations of human hypertension based on rats hypersensitive to a salt diet evidenced the substantial accumulation of extracellular matrix not during the initial remodeling phase of left ventricle hypertrophy, but throughout its transition toward congestive heart failure. This abnormal extracellular matrix organization is shown to be caused by the upregulation of endothelin I and angiotensin II genes (113–115).

However, the differences between rat strains (normotensive and spontaneously hypertensive)—and generally amid rodents and humans—substantiate animal models' incomplete suitability to recapitulate the complex pathologic events observed in clinical patients.

##### 2-D Cellular and 3-D Tissue-Engineered Models

*In vitro* mouse and human cellular models contribute to define the specific role and mutual effects of the different cell types participating in cardiac fibrosis. Activated cardiac fibroblasts increase the secretion of extracellular matrix in response to the paracrine signaling of cardiomyocytes, mechanically, or electrically stimulated (116, 117). Still, it is likely the amount of this first cell type but not the extent of the secreted extracellular matrix to induce the loss of contractility in the latter (118). Recently, Ibarrola et al. demonstrated for the first time through an *in vitro* model based on adult human cardiac fibroblasts and an *in vivo* murine model of mitral valve prolapse that the activation of the mineralocorticoid receptor contributes to myocardial fibrosis (119), thus identifying a valid target for drug treatment.

*In vitro* 3-D models of cardiac fibrosis combine several cell types (e.g., cardiac fibroblasts and myocytes) and scaffolds (collagen, gelatin methacryloyl hydrogels, etc.). By playing with the ratio between these two different tissue elements, they finely investigate the induced molecular and biomechanical changes (118, 120–122). These *in vitro* simulations provide evidence of the continuous crosstalk existing among involved cells in physiologic and pathologic conditions. The impairment of this crosstalk in myocardial fibrosis was revealed to be a consequence of the aging of fibroblasts (123). Stiffness typical of myocardial fibrosis can provoke the reactivation of extracellular matrix genes in cardiac myocytes (derived from iPSCs) (124) and might be rescued by cardiac progenitors (125). Besides the mechanistic studies, these models are predicted to show a strong efficacy in the developmental process of a finally effective, antifibrotic pharmacologic treatment.

Different cells, scaffolds, and conditioning can be included and modulated to reproduce at best all the possible variables observed *in vivo*. In particular, the use of patient-specific iPSCs could boost the development of a tailored antifibrotic medicine in a model taking as granted the genetic variants and, hence, the clinical subject's predisposition.

#### Fibrosis in Heart Valves and Arteries

The fibrotic process encountered in heart valves and arteries shares many similar aspects with that characterizing the myocardium but involves the interaction of different cells and extracellular matrix proteins. In heart valves, the cellular crosstalk is mainly established between endothelial cells and VICs, and in arteries, it interests predominantly the first cell type, together with fibroblasts and smooth muscle cells. Less represented cellular types, such as leukocytes, should be considered, too (126). In addition, both tissues display an extracellular matrix architecture that is physiologically more fibrous than in the myocardium. A substantial prevalence of collagen I is characterizing arteries and heart valves (127, 128). In this context, fibrosis is a pathological event often associated with others, such as stenosis or myxomatous degeneration in the heart valves and atherosclerosis in the arteries, separately described elsewhere in this review.

However, pure valvular and arterial fibrosis modeling is essential to understanding the single contribution given by this disease in a more composite pathological milieu. Only *in vitro* approaches can offer this possibility. Examples of arterial fibrosis modeling are reported in this section and in “Atherosclerosis,” while valvular fibrotic aspects are described in detail in “Cardiac aortic valve stenosis.”

##### 2-D Cellular and 3-D Tissue-Engineered Models

Typical signs of fibrotic arterial lesions (e.g., cell differentiation, proliferation, neointima formation) were accurately simulated *in vitro* with independent 2-D culturing of smooth muscle cells and explants of the aorta, both from rats, through the application of variable shear stress, intramural pressure, and/or vascular damage (129, 130).

#### Atherosclerosis

##### Epidemiology, Clinical Presentation, and Underlying Causes

The process leading to developing medium- and large-diameter atherosclerotic lesions in human arteries is multifaceted, complex, and progressive, involving many endogenous and exogenous players. Starting from the teenage period, it can progress and become manifest after 40–50 years. It is a disease widely spread among the worldwide population, especially in the Western industrialized countries, where a cholesterol-rich diet is the main culprit for its aetiogenesis (131). Women show a reduced tendency to develop atherosclerosis than men. Until the menopausal period, estrogen hormones exert an atheroprotective effect (132). Sex-dependent protection from atherosclerosis is negatively influenced by autoimmunity, which aggravates the inflammatory state characterizing this pathology (133).

An atheromatous plaque is an arterial lesion, which develops at the intimal layer. The atherosclerotic process starts with the injury of the intimal endothelial cells by several blood-related stimuli, such as pro-inflammatory cytokines, altered lipid content, and high pressure. Intimal damage induces an increase in permeability and activation of endothelial cells, thus allowing the arterial wall retention of low-density-lipoproteins (LDL) containing cholesterol. LDL arterial entrapment favors leukocyte adhesion and wall penetration. It also stimulates endocytosis by macrophages, transforming these cells into foam cells and contributing to plaque formation. Smooth muscle cells migrate from the media to the intima, attracted by chemokines released by activated cells, such as platelet-derived growth factor. This induces the switch of their phenotype from contractile to synthetic. An extracellular matrix enriched with collagen and elastin is secreted by migrated smooth muscle cells around the plaque, thus forming a fibrous cap. The extracellular space becomes crammed with cellular debris and partially digested lipids by dying macrophages, thus sharpening the inflammatory response in the plaque region. More robust recruitment and activation of inflammatory cells, especially macrophages, chronically contributes to the instability of the plaque by a boosted oxidative stress, an increased release of enzymes able to digest the extracellular matrix as well as chemokines inducing smooth muscle cell death or favoring thrombogenic events (19, 131, 134).

*Per se*, atherosclerosis is not a lethal condition. It alters the local shear stress physiological conditions, thus maintaining a state of activation in intimal endothelial cells. In addition, it limits the blood flow in the interested region due to generated arterial stenosis. The plaque may undergo an unstable evolution and rupture, resulting in the release of fragments into the bloodstream with thrombotic sequelae, such as myocardial infarct and stroke (19, 131, 134).

Essential efforts for (patho)physiology recapitulation and inclusion of all the risk factors accompany the mimicking of human atherosclerosis by *in vitro* and *in vivo* models. Differently from other mammalian species, humans are characterized by peculiar arterial anatomy with smooth muscle cells normally present in the intimal and medial layers as well as by a distinctive polarization pattern of inflammatory cells in innate and adaptive responses (19). Unhealthy lifestyles (e.g., smoke, fat-rich diet, insufficient physical exercise), preexisting pathological conditions (e.g., hypertension, diabetes mellitus), and genetic susceptibility are typically associated with human atherosclerosis onset and/or progression (131, 135). Atherosclerosis is undeniably one of the most modeled CVDs. Experimental approaches, mostly based on animals *in vivo*, have significantly contributed to a greater understanding of its pathomechanism although still far from full knowledge.

##### Animal Models

Mice, hamsters, rats, rabbits, guinea pigs, and lately pigs have been used as small or large models to investigate chronic atherosclerosis progression as previously reviewed (19, 136–140). In several animal models, species propensity to spontaneously develop atherosclerosis and/or feeding with cholesterol-rich diets have been exploited to induce or accelerate the development of atherosclerotic lesions in experimental settings. Through transgenesis strategies, it is possible to reproduce more efficiently the human disease phenotype in animal models, classically in mice, rats, rabbits, and in the last years in pigs, too (136, 138, 139, 141). Of note, hypercholesterolemia is almost the unique factor that has been considered in animal-based approaches (138).

Unlike humans, rabbits, and pigs, mice do not show a spontaneous predisposition to develop atherosclerosis. They are characterized by a different lipid gene regulation and metabolism with high-density lipoproteins (HDL) as the most critical cholesterol carrier (instead of LDL) (136). In addition, they possess a very definite immune polarization of the two populations of T helper lymphocytes (T_H_1 and T_H_2) and macrophages (M1 and M2) (19).

Due to easy handling and short lifetime, mice have been extensively used after transgenesis. Variants of human genes, recognized as associated with dyslipidemia and atherosclerosis, were introduced in the murine genome. Mice with defective apolipoproteins (e.g., apoE family) and LDL receptors reproduced hyperlipidemia, atherosclerosis onset, progression into advanced plaque, and complications. These models were also useful for monitoring disease evolution over time by applying knockout, insertional, or overexpression approaches and/or cholesterol feeding (142, 143). They demonstrated relevance for mechanistic and pharmacological studies to prevent or delay atherosclerosis. The drugs captopril, fosinopril, losartan, ramipril, and pravastatin were proved efficacious in mice, and nowadays, cardiology clinics apply them as an effective pharmacological treatment to lower hypercholesterolemia (144–147). Cutting-edge therapeutic strategies were also recently tested, such as microRNAs, nanoliposomes, and small interfering RNA nanoparticles to mitigate inflammation and smooth muscle cell migration (148–153). In addition, mouse transgenic models were useful to investigate the relationship between sex and atherogenesis, unraveling the protection from inflammation by testosterone but not from estrogen (154).

Rabbits are the second mammalian species mostly utilized in atherosclerosis modeling thanks to the higher phylogenetic and metabolic proximity to humans than mice. However, the reduced levels of hepatic lipase and hepatotoxicity after high-fat feeding, accompanied by the absence of plasmatic apoA-II and potent inflammatory response render this animal model dissimilar to humans and with more limited application than mice (137, 140). Inbreed selection of a Watanabe heritable hyperlipidemic strain with spontaneous hypercholesterolemia and genetic engineering for lipoprotein lipase, apoA-II, and hepatic lipase has partially overcome these limitations (137, 155–158).

Porcine models offer ideal conditions to reproduce human atherosclerosis in its many facets (arterial interest, intimal progression with foam cells and smooth muscle cells, high plaque vascularization with consequent hemorrhagic risk, calcification, and core necrosis). Atherosclerotic lesions in the pig do not advance spontaneously and supplementary triggers, as a hypercholesterolemic diet, are necessary. The high experimental cost can be a limiting factor in the use of the porcine model. As in the rabbit-based studies, these restrictions have been surpassed by inbreeding (159, 160), arterial mechanical damage (161), genetic engineering (161–164), and finally by the use of transgenic minipigs (163–165). By using engineered pigs, Pedigri et al. demonstrated that atherosclerotic lesion development is accelerated by chronically altered shear stress and is characterized by a thin cap fibroatheroma (164). Porcine atherosclerotic models are relevant to test new imaging devices and treatments, too (138, 166–169).

##### *In silico* Models

To dissect specific aspects of atherosclerosis, dual *in vitro* and *in silico* models by membrane bilayer reconstruction or cell conditioning are more affordable and suitable, being frequently applied to confirm observations coming from *in vivo* models. Membrane composition, particularly its phospholipid tail saturation and cholesterol quantity, profoundly impacts atherosclerotic plaque evolution. Bilayer models could potentially take into account atherosclerosis risk factors as well (170, 171).

Atherosclerotic pathophysiologic pathways are mainly studied and reconstructed in computational models. The genetic and genomic insights and knowledge gained by experimental animals and by affected patients are gathered together by several tools, such as genome-wide association studies. The latter depict the involved gene networks as well as the pathways that could be targeted to inhibit smooth muscle cell proliferation and stabilize atherosclerotic plaques (172, 173). Furthermore, comparative analyses of gene expression in other associated pathologies (e.g., stroke) and in the presence of risk factors (e.g., smoke) are allowing the discovery of diagnostic and prognostic markers of disease severity (173, 174).

All information collected by different atherosclerosis models was implemented in computational iterations to simulate the early and late stages of disease development in different scenarios of severity (175–177) and possibly may find use in the risk stratification of atherosclerotic patients.

##### 2-D Cellular Models

Cellular models were utilized alone or in tandem with *in vivo* approaches to confirm collected observations at the single-cell level. Endothelial cell lines, in particular derived from the human umbilical cord (HUVEC), serve mostly for this task to evaluate the effects of biochemical and/or mechanical damage, intended as first triggers for the development of the atherosclerotic lesion. These models of endothelial damage are useful to dissect the pathways that result in being dysregulated after contact with oxidative LDL (178, 179), with free fatty acids (180, 181), and in conditions of reduced shear stress or mechanical insult (182–184). Positive effects of atherosclerosis sign attenuation were disclosed by several *in vitro* studies using microRNAs on activated endothelial cells: miR-106a-5p, miR-144-5p, miR-200a, miR-490, and miR-500 (185–189).

Migration, acquisition of a synthetic phenotype, and proliferation represent pathological alterations of smooth muscle cell behavior during atherosclerosis. *In vitro* modeling using these cells aims at recapitulating these events for a clearer understanding of the underlying causes. Moreover, it intends to search for new prevention and intervention modalities, such as more accurate stenting procedures (190). In 1995, the group of Demer described at first the existence in the bovine arterial media of smooth muscle cells more prone to calcification and identified similar cell populations in human aortas (191). P38 mitogen-activated protein kinase, chemokine ligand 5 (CCL5)/chemokine receptor 5 (CCR5), and PPAR-γ gene signaling were among the pathways found to be involved in these smooth muscle cell alterations (192–195). *In vitro* pharmacological treatment with pyrogallol-phloroglucinol-6,6-bieckol from the brown alga *Ecklonia cava* reversed the altered phenotype of smooth muscle cells *in vitro* and *in vivo* (193). 17β-oestradiol-mediated upregulation of the PPAR-γ gene induced vascular protection in human coronary artery smooth muscle cells (195).

Patient-specific predisposition should also be considered in modeling atherosclerosis. Smooth muscle cells were differentiated from iPSCs derived from type 2 diabetes mellitus patients. They expressed higher arylacetamide deacetylase esterase levels when pharmacological protection from CVD was established. Metabolomic studies showed that these cells possessed fewer alterations in lipid metabolism and bioassembly. Esterase overexpression was confirmed to increase atheroprotection in primary smooth muscle cells as well as in apoE knockout mice (196).

#### 3-D Tissue-Engineered Models

Atherosclerotic shifts in blood flow dynamics affect endothelial function and platelet/leukocyte behavior. These effects are predicted to be recapitulated best by *in vitro*, 3-D bioengineered models (197–199). A circular microfluidic, endothelial cell–based stenosis platform was advanced by Venugopal Menon et al. By flow variations, the activated and dysfunctional state of endothelial cells and leukocytes is recapitulated and reversed by treatment with aspirin and metformin (200). Lv et al. develop a 3-D artificial vessel lined with endothelial cells to evaluate the cellular effects of stenting in a pulsatile flow system (201): restenosis and vasospasm were clearly identified as risks in these procedures by reactivation of endothelial cells.

Moreover, human bioengineered arterial equivalents could more accurately reproduce the dysfunctional interactions between the extracellular matrix and different cell types existing in atherosclerosis using bioreactors as a body surrogate and, thus, increasing the control over biological variables that could be associated with *in vivo* modeling. In 2013, Robert et al. advanced the first bioengineered model of atherosclerosis by submitting a tissue-engineered artery to LDL, HDL, and/or TNF alpha conditioning in the presence of circulating monocytes. Endothelial activation and increased permeability to lipoproteins were recapitulated. Monocytes transmigrated into the intimal layer without apparent evolution toward foam cells (26). The same group bioengineered a typical fibroatheroma by applying the hanging-drop technique (202). More recently, they developed a simple pulsatile flow system to recreate *in vitro* the specific flow conditions observed in aneurysms and atherosclerosis. By exposing bioengineered arteries to different velocity regimens, Hosseini et al. showed that no impairment of extracellular matrix genes (collagen I and elastin) was appreciable. They also revealed that the altered gene regulation of metalloproteinases and their tissue inhibitors reflected flow variations and could be reversed by doxycycline, already applied in the clinics for CVD treatment (203).

### Cardiac Aortic Valve Stenosis

#### Epidemiology, Clinical Presentation, and Underlying Causes

Cardiac disease or stenosis of the aortic valve (AVS) is the most frequently acquired valvular disorder in Western countries (127, 204) although also the mitral valve can be frequently affected (82). Its prevalence is continuously rising, especially among the aging population. Currently, no effective pharmacological therapy can slow down the progression of this chronic disease, which becomes symptomatic only after reaching clinical severity and high mortality risk (205). BAV-affected patients have an increased tendency to develop AVS. More than 300,000 valve replacement procedures are performed annually as a unique treatment for end-stage valvular disease (24, 206) to restore physiologic blood flow. An AVS echocardiographic sign is a valvular jet velocity reaching 4.7 m/s, and slower values of 2.4 m/s, although altered, indicate sclerosis. Upon histopathological examination, a stenotic aortic heart valve appears to be composed of evident lipocalcific deposits in the fibrosal aortic cusp aspect and at the commissures (207). These tissue alterations are responsible for strongly reduced leaflet mobility.

The AVS process has been considered in parallel to arterial atherosclerosis due to the pathomechanistic shreds of evidence (208). Similar hallmarks are injury and activation of endothelial cells, permeability and deposition of lipids, monocyte/macrophage recruitment, differentiation of cells (VICs in this case) toward a myo/calcific phenotype, neovascularization, and tissue mineralization. However, smooth muscle cells are not involved, and calcification occurs earlier in stenotic aortic valves than in atherosclerotic arteries. In addition, no influence of diabetes is found to be particularly relevant for AVS development and/or progression (207). Moreover, specific genetic predisposition to AVS has been demonstrated as associated with apolipoprotein B and (a), angiotensin-converting enzyme, and IL10 gene polymorphisms (209).

It is still poorly understood how a sclerotic valve may become stenotic. Nevertheless, the recurrence of impaired gene expression, extracellular matrix dysregulation, altered hemodynamics, and coexisting risk factors are considered as possible causes. In particular, an endMT program reactivation characterized by altered NOTCH1 gene pathway regulation in valve endothelial cells is hypothesized as the first event of the pathologic molecular cascade leading to AVS (210). Lipid accumulation starts when activated valve endothelial cells increase permeability, and intra-intimal transport by apolipoproteins B and E is facilitated (211).

#### *In silico* Models

For risk stratification and health care management, AVS computational modeling, especially of patient-specific disease, offers new strategic insights. Adda et al. used an *in vitro* mock circulatory system to investigate different scenarios of AVS severity, stroke volume, heart rate, and mean arterial pressure. Their study evidenced that a stenotic valve impairment with effective orifice area <0.85 cm^2^ has to be considered severe with a mean gradient ≥25 mmHg in conditions of low flow or ≥37 mmHg in the presence of normal flow (212). The modeling of the fluid interactions of AVS patients by Kivi et al. revealed that the transvalvular pressure gradient increases by 3.6-fold from healthy to severely calcified valves with a consequent 2.2-fold decrease of blood velocity in coronary arteries at early diastole (213). Bhuva et al. applied machine learning to study sex and regional differences in myocardial plasticity due to AVS. Cardiovascular magnetic resonances of AVS patients were collected before and after 1 year surgery. Data were used to develop a 3-D model of wall thickness, showing that remodeling was more pronounced in the septum of male patients both pre- and post-surgery (17).

#### Animal Models

In atherosclerotic mouse models based on defective apolipoproteins, the calcific involvement of the aortic valve beside the coronary arteries is usual (214). Cusp thickening and stenosis might be observed only after lipopolysaccharide administration, generally introduced to enhance inflammatory response (215). In order to reproduce more effectively all the clinical signs of human AVS in mice, Niepmann et al. proposed the use of a novel model developed with coronary wires altering the shear stress at the cusp valve endothelium (216).

Alongside the widely used mouse, larger animals also served as *in vivo* platforms to study AVS evolution. Cuniberti et al. used a New Zealand white rabbit model of hypertension to evaluate the link between this risk factor and AVS (217). Sider et al. replicated the first events of heart valve sclerosis through a 5 month-long hypercholesterolemic model based on Yorkshire pigs. Lipid accumulation and proteoglycan-enriched extracellular matrix but no myofibroblast phenotype and calcification were observed (218).

#### 2-D Cellular Models

*In vitro* cellular models have provided much understanding of the altered pathological pathways in AVS. As mentioned, valve endothelial cells are the first player in the sclerotic valve cusp. Still, the central role in AVS development is exerted by the heterogeneous cell population of VICs, which, upon stimulation, can acquire a myofibroblast phenotype and ultimately switch to calcifying cells.

Semilunar and atrioventricular heart valve fibrosis models were initially created in two dimensions. By reproducing transvalvular pressure and/or TGF-ß biochemical stimulation, they allowed clarifying their effects on VICs. In particular, increased cell activation was observed as prompted by a stiffer extracellular matrix (219). A more substantial susceptibility was shown for VICs derived from the left inflow and outflow tracts (220), thus reflecting the significant involvement of the aortic and mitral valves observed in the clinic. Furthermore, endMT in involved valve endothelial cells strongly potentiated VIC activation (221).

Analogous to arterial disease (191), calcification-prone cell populations have been identified in the *fibrosa* of canine, bovine, and human aortic valve cusps by tissue explant, cell cloning technique, and *in vitro* calcification modeling (222, 223). The conditioning with TGF beta-1, 25-hydroxycholesterol, and/or bone morphogenetic protein 2 stimulated in nearly 1 month a massive generation of calcified nodules in canine VICs (222). Upon *in vitro* exposure to lipopolysaccharide (LPS) for 12 days, clones of bovine VICs displayed different responses with only one osteocalcin-positive cell type undergoing calcific switch after expressing higher levels of alkaline phosphatase (223). The proteomic analysis on these conditioned VIC subpopulations showed alterations in the expression of nearly 50 cytosolic and membrane-associated proteins. Modifications were observed after LPS conditioning in several functions: chaperone-mediated protein folding, protein metabolism and transport, cell redox/nitric oxide homeostasis, cytoskeletal organization, and nitric oxide bioactivity. In particular, tackling the nitric oxide pathway by controlling the L-arginine/ADMA ratio through L-arginine administration was efficient to prevent calcification (224). In addition, conditioning with natural biomolecules pyrophosphate and ATP was sufficient to strongly inhibit LPS-mediated mineralization of calcifying clonal VICs in 2-D and 3-D cultures (225). As LPS, other pro-inflammatory stimuli have been shown to induce VIC calcification. Warnock et al. exposed porcine aortic valve cusps to high cyclic pressures. They verified altered transcription for genes related to inflammatory responses, such as the cytokines TNF-α, IL-1α, and IL-1β, as well as acute phase protein pentraxin 3 and also metalloproteinases (226).

Microenvironment sensing is a highly developed ability in VICs. These cells sense the extracellular matrix modifications and, consequently, respond by varying their gene expression. RhoA/ROCK and PI3K/AKT pathways are the most activated ones in response to stiffness. Upon their upregulation, VICs differentiate into activated myofibroblasts, which are highly proliferative, secreting extracellular matrix, and enriched with smooth muscle actin stress fibers (227).

The evidence that NOTCH1 repression suppresses VIC calcification comes from a previously described study by Nigam and Srivastava (39). They observed no occurrence of calcification in sheep VICs after the inhibition of the bone morphogenetic protein 2 downstream target genetic pathway through siRNA-mediated knockdown.

#### 3-D Tissue-Engineered Models

As for modeling other CVDs, 3-D VIC-based systems are valuable tools to reproduce the complex pathophysiologic events occurring in calcific aortic valve cusps (225, 228–230). Mabri et al. showed that increasing the matrix modulus of poly(ethylene glycol) hydrogels induced VICs' phenotypic acquisition of smooth muscle actin stress fibers (231). Hjortnaes et al. established a 3-D platform recapitulating the early AVS phases. Osteogenic stimulation was provided to valve tissue constructs generated by coupled hydrogels and aortic VICs. The latter underwent differentiation to smooth muscle actin- and Runx2-expressing myofibroblasts and finally calcification. Mineralization could be reduced by silencing smooth muscle actin gene expression, thus leading to hypothesize myofibroblast differentiation as the first step in AVS calcification (229). In the study by Duan et al. 3-D hydrogels with tunable stiffness were populated with VICs and submitted to osteogenic media. Once again, a dynamic feedback loop was identified, demonstrating the essential role of the mechanosensitive RhoA/ROCK pathway in calcification (228). Dahal et al. speculated on the role of glycosaminoglycans on AVS pathologic endMT through modular tissue reconstruction (221). Collagen scaffolds supplemented with chondroitin sulfate, hyaluronic acid, and dermatan sulfate simulated early- and late-stage AVS phases. The modeling with chondroitin sulfate-enriched scaffolds correlated with enhanced endMT and increased extracellular matrix synthesis. The impact of a more complex extracellular matrix on VIC behavior has also been considered by Monroe et al. Using laminar constructs with variable composition, they noted reduced cell survival and increased collagen secretion in mineralizing cells (230). More recently, hydrogels derived from decellularized valve extracellular matrices were used as scaffolds for 3-D AVS modeling (232), revealing superiority for cell growth and proliferation over pure collagen.

AVS therapeutic could be complex with previous modeling modalities, but pathology bioengineering might help in the testing and fast release of new treatments. Targeting the typical AVS microRNA dysregulation could be a valid strategy to stop disease progression as recently reviewed by van der Ven et al. (233). For example, miR-34c was confirmed in a 3-D bioengineered AVS model to inhibit VICs' osteogenic differentiation through c-JUN terminal kinase pathway suppression (234). As observed in 2-D VIC cultures, Weber et al. proved the involvement of purinergic signaling in AVS through their 3-D models (235). Potentially, a novel pharmacological compound could be identified to lower adenosine levels and, hence, protect from valve degeneration.

## Closing Remarks

*In silico, in vivo*, and *in vitro* models have unquestionably helped increase the comprehension of CVDs, propose underlying molecular mechanisms, and/or test novel therapeutic hypotheses. More and more, cardiovascular research is moving toward integrating the different approaches applied so far to overcome their own limitations and inabilities to fully reproduce the human pathophysiology of the heart and its system. Along this direction, the advent of frontline technologies, such as human iPSCs and tissue engineering, represent a decisive milestone to bridge these gaps. The ability to reconstruct high-fidelity equivalents of human tissues and organs *in vitro* by applying bioengineering principles has started to demonstrate its validity to decipher the healthy and pathologic interactions among cells and extracellular matrix. The recapitulation of this functional crosstalk and its impairment through tissue engineering provides novel or more specific insights on the molecular pathways involved in CVDs and could be addressed as therapeutic targets. These *in vitro* platforms appear to be highly modular tools. A unique possibility for a personalized approach of precision medicine is offered by playing with defective extracellular matrices (e.g., increasing the stiffness of synthetic hydrogels or using decellularized pathologic cardiovascular tissue), patient-specific cells (e.g., differentiated iPSCs harboring a genetic mutation or a peculiar polymorphism), and/or with their differential ratio. Diseases, such as CPVT, AC, atherosclerosis, cardiovascular fibrosis, and AVS, have already been reproduced *in vitro* through a bioengineering approach. The study of BAV and cardiac infections—and other pathologies not considered in this review—could positively take advantage of tissue engineering–based modeling. For example, the emergent property of biofilm formation, shown by some bacterial species on cardiovascular mucosae, could be modeled by recreating in a Petri dish the endo-myocardial layers of the ventricular wall and bacterial contamination. Such a simulation could possibly allow proceeding into the search of modalities for biofilm dissolution and tackle a medical threat, such as bacterial multidrug resistance, especially in nosocomial infections.

Besides this, tissue-engineered human models of cardiovascular pathologies could serve as reliable platforms to verify the mechanistic and therapeutic predictions on CVDs, generated by inference from coupled -omics technologies and machine learning.

These bioengineered platforms are just showing the first signs of potential into effective disease modeling. They are predicted to bring an incredible advancement in patient-tailored pharmacological strategies and health care management improvement for both congenital and acquired CVDs.

## Author Contributions

LI personally contributed to the conception and writing of the manuscript.

## Conflict of Interest

The author declares that the research was conducted in the absence of any commercial or financial relationships that could be construed as a potential conflict of interest.
